# Using cerebrospinal fluid to confirm *Angiostrongylus cantonensis* as the cause of canine neuroangiostrongyliasis in Australia where *A. cantonensis* and *Angiostrongylus mackerrasae* co-exist

**DOI:** 10.1016/j.crpvbd.2021.100033

**Published:** 2021-06-01

**Authors:** Jeevitheswara Thammannaya Mallaiyaraj Mahalingam, Nichola Eliza Davies Calvani, Rogan Lee, Richard Malik, Jan Šlapeta

**Affiliations:** a Sydney School of Veterinary Science, Faculty of Science, University of Sydney, 2006, New South Wales, Australia; b Molecular Parasitology Laboratory, Centre for One Health Ryan Institute, National University of Ireland, Galway, H91 DK59, Galway, Ireland; c Parasitology Laboratory, Centre for Infectious Diseases and Microbiology Lab Services, NSW Health Pathology, Level 3 ICPMR, Westmead Hospital, 2145, New South Wales, Australia; d Centre for Veterinary Education, University of Sydney, 2006, New South Wales, Australia

**Keywords:** Rat lungworm, Dogs, Haplotype, Validation, CSF, Mitochondrial DNA, Molecular diagnostics

## Abstract

Both *Angiostrongylus cantonensis* and *Angiostrongylus mackerrasae* have been identified along the east coast of Australia. A lack of *A. mackerrasae* genomic data until 2019, however, has precluded the unequivocal identification of the *Angiostrongylus* species responsible for neuroangiostrongyliasis in accidental hosts such as dog and man. The availability of a whole-genome data for *A. mackerrasae*, including mtDNA and ITS2 rDNA, enables discrimination of *A. cantonensis* from *A. mackerrasae*. The aim of this study was to develop diagnostic PCR assays to determine the species of *Angiostrongylus* based on the detection of *Angiostrongylus* DNA sequences in the cerebrospinal fluid (CSF) of canine patients with eosinophilic meningitis. An *in silico* workflow utilising available cytochrome *c* oxidase 1 (*cox*1) primers streamlined the laboratory work into empirical steps, allowing optimisation and selection of a PCR assay that met the required criteria for discrimination of *A. cantonensis* and *A. mackerrasae* DNA in low-template CSF samples. The adopted *cox*1 qPCR assay specifically amplified and enabled the differentiation of *A. cantonensis* from *A. mackerrasae* DNA and confirmed the presence of *A. cantonensis* DNA in 11/50 archived CSF samples. The DNA sequences demonstrated the presence of two distinct *A. cantonensis cox*1 haplotypes in dogs from eastern Australia. Species identification was further confirmed *via* the adoption of an ITS2 rDNA assay, providing confirmation of only *A. cantonensis* ITS2 rDNA in the CSF samples. To our knowledge, this is the first study to unequivocally demonstrate the antemortem presence of *A. cantonensis* DNA in CSF from clinically affected dogs. The study confirmed the long-held assumption that *A. cantonensis* is the causal agent of neuroangiostrongyliasis but refutes the dogma that there was a single introduction of *A. cantonensis* into Australia by the demonstration of two distinct *A. cantonensis cox*1 haplotypes.

## Highlights

• Adaptation of existing *cox*1 and ITS2 rDNA PCR assays detected *Angiostrongylus* DNA in the cerebrospinal fluid (CSF) of canine patients.
• An *in silico* workflow enabled streamlined empiric laboratory steps for *cox*1 PCR optimisation to discriminate *Angiostrongylus mackerrasae* and *Angiostrongylus cantonensis.*
• *A. cantonensis* but not *A. mackerrasae* was demonstrated in CSF specimens from clinically affected dogs.
• The presence of two distinct *A. cantonensis cox*1 haplotypes was identified, suggesting multiple introductions into Australia.

## Graphical abstract

**Figure:**
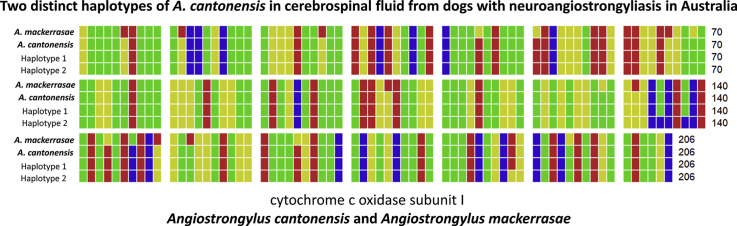

## 1 Introduction

The rat lungworm (*Angiostrongylus cantonensis*) is a nematode parasite of rats and gastropod molluscs known to accidently infect humans, dogs, and other animals where it causes eosinophilic meningitis and neuroangiostrongyliasis (NA) (Wang et al., 2008; Lee et al., 2021). Alongside its definitive hosts, rats in the genus *Rattus*, *A. cantonensis* has invaded many regions of the world including Australia (Mackerras & Sandars, 1955; Tokiwa et al., 2013; Rodpai et al., 2016). Humans are exposed to *A. cantonensis via* the consumption of snails and slugs, or by the ingestion of paratenic hosts such as crabs, prawns and planarians (Wang et al., 2008). Once ingested, larval stages of *A. cantonensis* have an obligatory migration through the central nervous system (CNS), where a florid immunological response in accidental hosts leads to syndromic clinical presentations (Murphy & Johnson, 2013).

Numerous cases of NA have been recorded in humans and animals along the east coast of Australia over the past 50 years (Bhaibulaya, 1968; Wang et al., 2008; Diao et al., 2011; Lunn et al., 2012; Murphy & Johnson, 2013), with the most renowned case being that of a Sydney teenager who was infected after eating a slug on a dare (Senanayake et al., 2003). In Australia, dogs are much more commonly infected with *Angiostrongylus* spp. than people, and yet there are limited methods available for the diagnosis of canine NA. Diagnosis is contingent on the invasive collection of cerebrospinal fluid (CSF) and demonstration of eosinophilic pleocytosis (Lunn et al., 2003; Lee et al., 2021). Further confirmation requires either ELISA testing for anti-*A. cantonensis* antibodies and, more recently, the confirmation of *Angiostrongylus* spp. DNA *via* qPCR (Lee et al., 2021).

As a generalisation, NA is assumed to be caused by infection with *A. cantonensis* (Barratt et al., 2016). In Australia, however, two *Angiostrongylus* species – the invasive emerging *A. cantonensis* and the native *A. mackerrasae* – are known to be neurotropic in their definitive rat hosts (Bhaibulaya, 1974; Červená et al., 2019; Valentyne et al., 2020). A lack of methods enabling species differentiation has prevented the definitive confirmation or exclusion of *A. mackerrasae* as a cause of clinical disease in dogs. At least one report using morphology demonstrated the presence of patent *A. mackerrasae* as the cause of angiostrongyliasis in a black flying fox (Mackie et al., 2013). Aside from rare instances where necropsy material has enabled species differentiation, the causative agent of NA in dogs in Australia remains unresolved (Lunn et al., 2012; Lee et al., 2021).

Recently, the species status of *A. cantonensis* and *A. mackerrasae* was re-confirmed using complete mitochondrial (mt) DNA, where a 7.1–7.5% difference between the two was demonstrated (Valentyne et al., 2020). The molecular discrimination of *Angiostrongylus* spp. commonly employs mtDNA, with many studies targeting cytochrome *c* oxidase 1 (*cox*1) from either adult specimens or larval stages within gastropod molluscs (Tokiwa et al., 2013; Lv et al., 2017, 2018). We hypothesise that the high relative abundance of *cox*1 template DNA available for amplification, combined with the significant nucleotide difference between *A. cantonensis* and *A. mackerrasae* (∼10%), may enable the differentiation of *Angiostrongylus* spp. *cox*1 DNA sequences in cerebrospinal fluid (CSF) in suspected cases of canine NA and hence confirm the causative agent of disease.

The aim of this study was to adapt existing primers to develop a PCR assay that can determine the species of *Angiostrongylus* present in the CSF of canine patients based on the detection of mtDNA. To do so, we utilised archived and curated CSF samples from canine NA cases from Australia (Lee et al., 2021). The PCR assay targeted the highly variable *cox*1 region, which is able to discriminate reference *A. cantonensis* DNA from that of *A. mackerrasae* (Valentyne et al., 2020). The *cox*1 DNA sequence results were further verified by the adoption of a nuclear marker enabling DNA sequence analysis of ITS2 rDNA assay, which was capable of amplifying both *A. cantonensis* and *A. mackerrasae*.

## 2 Materials and methods

### 2.1 Samples

DNA previously extracted from *A. cantonensis* (SYD.1, 10 ng/μl) and *A. mackerrasae* (ANWC:N5721 - P43/19-E, 2 ng/μl) were used throughout the study as positive controls, while DNA from archived CSF samples of 61 dogs from eastern Australia with eosinophilic meningitis (DOG 1-61, 50 PCR-positive, 11 PCR-negative but antibody-positive) were used for genotype screening (Červená et al., 2019; Valentyne et al., 2020; Lee et al., 2021). As a negative control, canine DNA was extracted from whole dog blood using Monarch DNA Isolation kit (New England Biolabs, Australia) using the manufacturer's instructions. Two additional CSF samples from dogs with canine NA were included and DNA was isolated using Isolate II DNA isolation kit (BioLine, Australia). Canine blood and CSF samples were sourced from the molecular diagnostic laboratory at Veterinary Pathology and Diagnostic Services (VPDS), University of Sydney. DNA was stored at −20 °C prior to PCR testing.

### 2.2 *In silico* selection of *cox*1 primers to amplify *A. cantonensis* and *A. mackerrasae*

Mitochondrial markers from complete Australian reference mtDNA genomes of *A. cantonensis* SYD. 1 (GenBank: MK570631) and *A.* mackerrasae P43/19-E (GenBank: MN793157) were used to compare the similarity of mitochondrial regions (Červená et al., 2019; Valentyne et al., 2020). All *cox*1 primers from previous studies on *Angiostrongylus* parasites were collated and tabulated (Monte et al., 2012; Tokiwa et al., 2012; Moreira et al., 2013; Nakaya et al., 2013; Okano et al., 2014; Apichat et al., 2016; Rodpai et al., 2016; Eamsobhana et al., 2017; Červená et al., 2019; Valentyne et al., 2020). Primers were mapped onto the reference sequences of *A. cantonensis* SYD. 1 (MK570631) and *A. mackerrasae* P43/19-E (MN793157) and the number of mismatches and their position relative to the reference mtDNA genomes were recorded before a combination of forward and reverse primers were selected for testing. Primers met the following criteria: (i) amplification of a short sequence (< 300 bp) that (ii) enabled species differentiation between *A. cantonensis* and *A. mackerrasae* with (iii) ≤ 3 mismatches between the primer and template DNA sequence (Table 1).

**Table 1:** Primers used in this study to amplify *Angiostrongylus* spp. DNA

Primer name	F/R	Primer sequence	Position	No. of *Angiostrongylus cox* 1 mismatches	
				*A. mackerrasae*	*A. cantonensis*
cox1F [S0962]	F	TTTGTTTTGATTTTTTGGTC	720–739	1	0
AngiCOI_forward [S0963]^a^	F	TTTTTTGGGCATCCTGAGGTTTAT	730–753	2	1
LCO1490	F	GGTCAACAAATCATAAAGATATTGG	47–71	8	9
AC1F [S0964]	F	CGGGTAAGAAGGAGGTTTTTG	806–826	0	2
AC2F [S0965]	F	AGTTATTGCGGTTCCTACGG	951–971	0	1
AC1R [S0966]	R	CCTTCACTCCCGTAGGAACC	960–979	0	2
AC2R [ S0967]	R	TTAGACAACATAACCCCAGTCAA	1078–1100	1	2
HCO2198	R	TAAACTTCAGGGTGACCAAAAAATCA	727–752	1	2
COI_R^b^	R	TAAAGAAAGAACATAATGAAAATG	1147–1170	6	6
cox1R	R	AGGATAAATCTAAATACTTACGAGGA	1329–1354	7	6
AngiCOI_reverse	R	CGAGGATAACCATGTAAACCAGC	1312–1334	2	2

*Note*: Sequences of forward (F) and reverse (R) *cox*1 primers used in previous studies and their position relative to reference sequences *A. cantonensis* (GenBank: AMK570631) and *A. mackerrasae* (GenBank: MN793157) are shown (Apichat et al., 2016; Červená et al., 2019; Eamsobhana et al., 2017; Monte et al., 2012; Moreira et al., 2013; Nakaya et al., 2013; Okano et al., 2014; Rodpai et al., 2016; Tokiwa et al., 2012; Valentyne et al., 2020).

a Also named ‘COI_F’, ‘CO1_R’, ‘239’, ‘2575’.

b Also named ‘CO1_F’, ‘240’, ‘3021’.

### 2.3 Partial *cox*1 PCR assay selection for the detection of *A. cantonensis* and *A. mackerrasae* DNA

Assays selected *in silico* were then tested using conventional PCR on 10-fold serial dilutions of isolated *A. cantonensis* DNA (SYD.1) from 200 to 0.2 pg/μl. PCR amplifications were made up to 30 μl, including 2 μl of template DNA, and were performed using MyTaqTM Red mix (Bioline, Australia), distilled water and primers at a concentration of 0.33 μM. Each PCR run included a no template negative control (distilled water). Cycling conditions used were as follows: 95 °C for 3 min, followed by 35 cycles of 95 °C for 15 s, 52 °C for 15 s and 72 °C for 20 s, with a final extension step at 72 °C for 7 min. Selected *cox*1 assays capable of amplifying the highest dilution of *A. cantonensis* DNA (0.2 pg/μl) were then optimised using a temperature gradient from 52 °C to 62 °C, as described above, to decrease the presence of non-specific binding and primer dimers. Finally, the selected assays were tested on *A. mackerrasae* DNA isolated from an adult worm and dog DNA to determine the ability of the primer pairs to amplify both species. Two additional known-positive canine CSF samples (see Section 2.1) were included to ensure their capacity to detect *Angiostrongylus* spp. DNA in the presence of canine eosinophils. PCR products were visualised on a 2% agarose gel stained with GelRed (Biotium) and observed under UV-light.

### 2.4 Detection of *Angiostrongylus* spp. *cox*1 DNA *via* qPCR from the CSF of dogs with eosinophilic meningitis

The DNA from CSF samples of 61 dogs from eastern Australia with eosinophilic meningitis were screened using the selected *cox*1 assay *via* real time PCR (qPCR); of these, 50 dogs were previously confirmed PCR-positive for *Angiostrongylus* spp. DNA using a highly sensitive qPCR *sensu* Sears et al. (2021), while 11 were PCR-negative but antibody-positive (Lee et al., 2021). The partial *cox*1 qPCR reaction mixtures were made up to 20 μl, including 2 μl template DNA, using Luna Universal qPCR Mastermix (New England Biolabs, Australia) and contained primers at a final concentration of 0.25 μM. The qPCR reactions were run on the CFX96 Touch Real-Time PCR Detection System (BioRad, Australia) and analysed using the corresponding CFX Maestro 1.0 software (BioRad, Australia). Cycling conditions were as follows: 95 °C for 60 s, followed by 40 cycles at 95 °C for 15 s and 55 °C for 30 s. The cycling protocol was finished with a melt curve cycle. Each qPCR run contained reference *A. cantonensis* and *A. mackerrasae* DNA from adult nematodes as the positive controls and distilled water as a no-template negative control. Results were considered positive if the melt curve profile corresponded to that of either of the positive controls. Samples returning C_t_ values < 40 with expected melt curves were submitted for bidirectional sequencing at Macrogen Inc. (Seoul, Korea). DNA chromatographs were inspected for quality and ambiguity by eye and aligned to reference mtDNA genome sequences, *A. cantonensis* SYD.1 (MK570631) and *A. mackerrasae* P43/19-E (MN793157) (Červená et al., 2019; Valentyne et al., 2020). A reference alignment of all known *cox*1 sequences was compiled from GenBank with the aid of nblast. Newly obtained sequences were appended to the reference alignment and percentage identity to the known *cox*1 haplotype was evaluated.

### 2.5 Detection of *Angiostrongylus* spp. ITS2 *via* qPCR from the CSF of dogs with eosinophilic meningitis

As a nuclear marker, the second ribosomal transcribed spacer sequence ribosomal DNA (ITS2) from *Angiostrongylus* spp. was targeted using a dual labelled probe AC (5′-FAM-GCT ACA TGT AAT AAT TCG ACG ATA TGT G-BHQ-3′) and two primers FP (5′-CCA GTT TTG GTG AAG AAT AA-3′) and RP (5′-ACA CGA CGG TAA CAA TGA CA-3′) amplifying ∼140 bp product (Fang et al., 2012). The DNA from the CSF samples from the 61 dogs described in Section 2.4*.* were screened. The ITS2 qPCR reaction mixtures were made up to 20 μl, including 2 μl template DNA, using Luna Universal Probe qPCR Master Mix (New England Biolabs, Australia) and contained primers and probe at a final concentration of 0.25 μM and 0.2 μM, respectively. The qPCR reactions were run on the CFX96 Touch Real-Time PCR Detection System (BioRad, Australia) and analysed using the corresponding CFX Maestro 1.0 software (BioRad, Australia). Cycling conditions were as follows: 95 °C for 180 s, followed by 40 cycles at 95 °C for 5 s and 60 °C for 15 s. Each qPCR run contained reference *A. cantonensis* and *A. mackerrasae* DNA from adult nematodes as the positive controls and distilled water as the no-template negative control. Samples returning C_t_ values < 40 were submitted for bidirectional sequencing at Macrogen Inc. (Seoul, Korea). DNA chromatographs were inspected for quality and ambiguity by eye and aligned to reference ITS2 sequences, *A. cantonensis* SYD.1 and *A. mackerrasae* P43/19-E, assembled from next generation sequence data (Červená et al., 2019; Valentyne et al., 2020).

## 3 Results

### 3.1 *In silico* selection of *cox*1 primers to amplify *A. cantonensis* and *A. mackerrasae*

The *cox*1 region was selected as the mitochondrial marker for further interrogation based on the high percentage difference between the *cox*1 sequences (9.1%) of *A. cantonensis vs A. mackerrasae*, as well as the variety of *cox*1 primers available (*n* = 11) for *Angiostrongylus* spp. (Table 1). Five combinations of four reverse and two forward primers capable of differentiating *A. cantonensis* and *A. mackerrasae* were selected to minimise the number of primer-template mismatches (≤ 3) and meet the desired amplicon length (< 300 bp): Assay 1: ‘cox1F & AC1R’ primers which amplify a 259-bp region, in the position 720–979 relative to the reference *cox*1; Assay 2: ‘AngiCOI_forward & AC1R’ primers which amplify a 249-bp region, in the position 730–979; Assay 3: ‘AC1F & AC2R’ primers which amplify a 294-bp region, in the position 806–1100; Assay 4: ‘AC1F & AC1R’ primers which amplify a 173-bp region, in the position 806–979; Assay 5: ‘AC2F & AC2R’ primers which amplify a 149-bp region, in the position 951–1100.

### 3.2 Testing of different dilutions of *A. cantonensis* DNA to select the appropriate assay

**Fig. 1:**
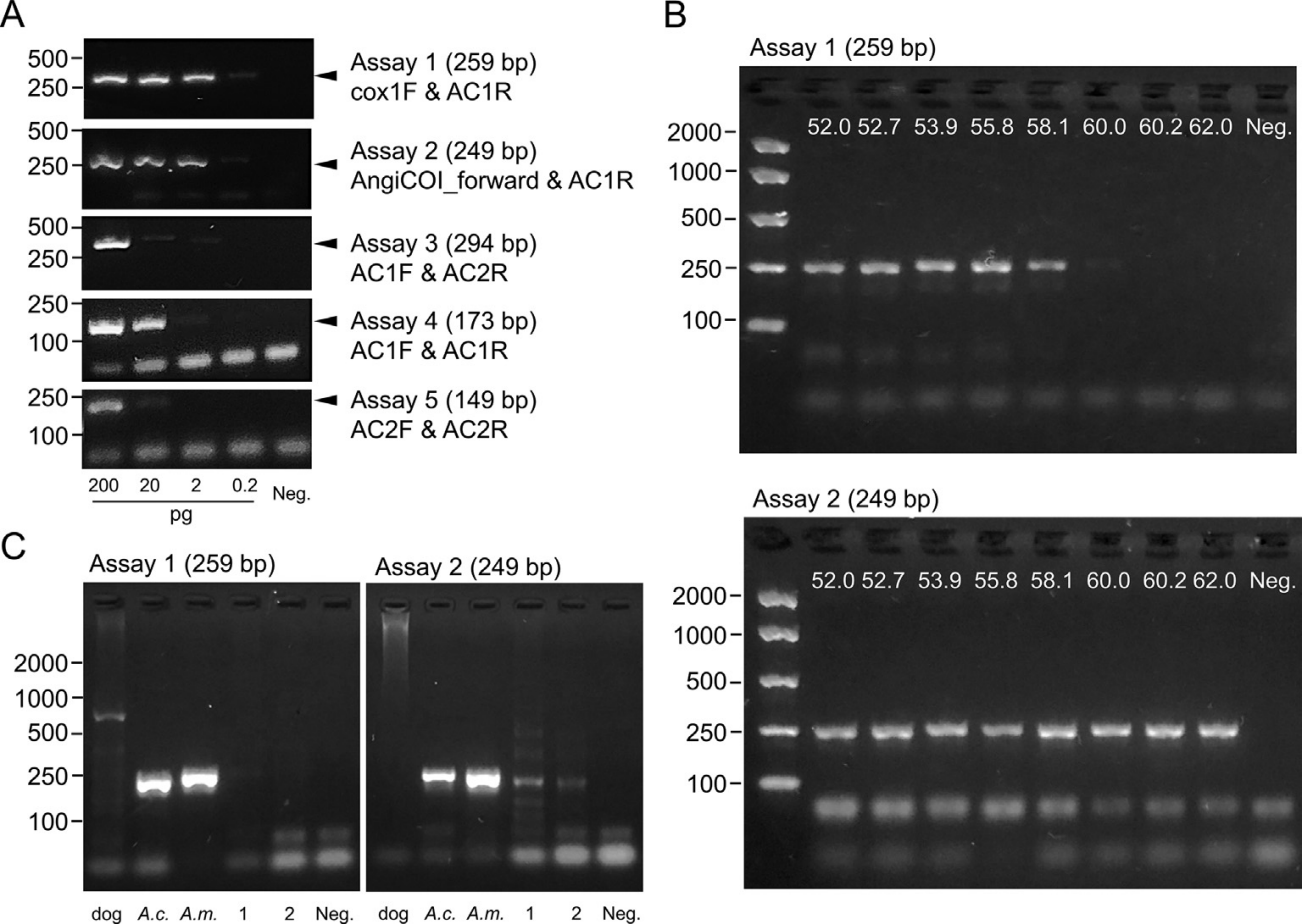
Amplification of *Angiostrongylus* spp. DNA using primers targeting partial *cox*1 mtDNA. **A** Five assays were tested on a 10-fold serial dilution of *A. cantonensis* (SYD.1) DNA extracted from a voucher specimen using an annealing temperature of 52 °C. For each assay, the respective primers and expected amplicon size are indicated to the right of the gel. The black arrow indicates the expected *Angiostrongylus* spp. *cox*1 mtDNA amplicon. **B** A temperature gradient PCR for Assay 1 and Assay 2 demonstrating specific amplification (∼250 bp) of *A. cantonensis* DNA as well as the existence of some low molecular weight (potential primer-dimer) non-specific amplification. **C** Demonstration of the ability of Assay 1 and Assay 2 to amplify both *A. mackerrasae* (*A.m.*) and *A. cantonensis* (*A.c.*) mtDNA using an annealing temperature of 55 °C. Note that Assay 1 amplified a ∼800-bp non-specific product from pure canine DNA (300 ng). Assay 2 was able to amplify the expected ∼250-bp *Angiostrongylus cox*1 amplicon from two known-positive canine CSF DNA samples (1, 2). All products were run on 2% agarose gels stained with GelRed and visualised under UV-light

All five *cox*1 assays produced a single amplicon of the expected size from *A. cantonensis* DNA (Fig. 1A). Assay 1 and Assay 2 amplified the highest dilution of *A. cantonensis* DNA (0.2 pg/μl) and were thus tested using a temperature gradient between 52 °C and 62 °C, respectively, to increase specificity and reduce primer dimer formation (Fig. 1B). Amplification of *A. cantonensis* DNA only occurred when the annealing temperature was ≤ 58 °C for Assay 1, while Assay 2 produced specific bands across the full temperature range. While amplification of *A. mackerrasae* DNA was achieved using both Assay 1 and Assay 2 primer sets, a ∼700-bp nonspecific dog DNA product was produced by Assay 1 at 55 °C (Fig. 1C). The successful amplification of DNA from two selected known-positive canine CSF samples was achieved by Assay 2 but not Assay 1 at 55 °C and thus Assay 2 was chosen as the preferred assay for the amplification of *Angiostrongylus* DNA in canine CSF samples for further adaptation using qPCR to enable medium throughput sample processing.

### 3.3 Two distinct *cox*1 mtDNA *A. cantonensis* haplotypes are present in canine CSF samples from eastern Australia

**Fig. 2:**
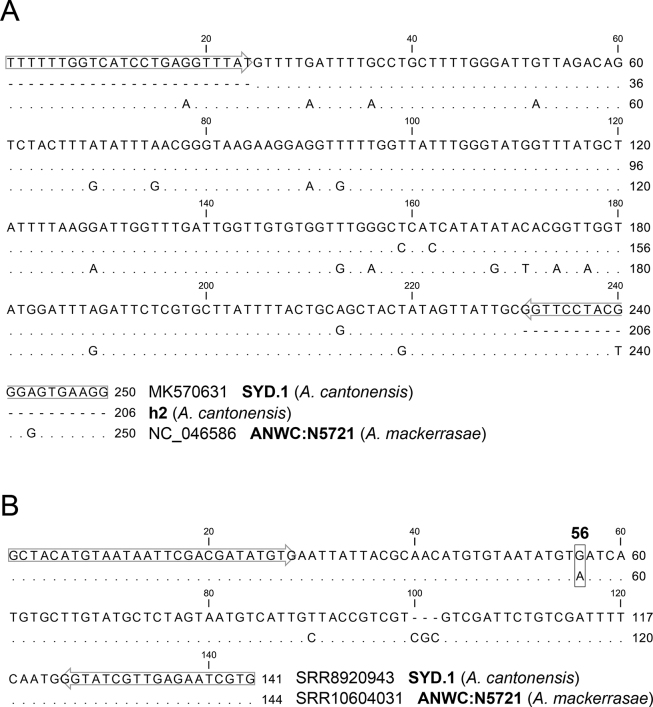
Multiple sequence alignment of the *Angiostrongylus* spp. DNA sequences amplified from canine CSF samples. **A***A. cantonensis* (SYD.1) and *A. mackerrasae* (ANWC:N5721) *cox*1 mtDNA and the novel haplotype 2 (h2) amplified using Assay 2. **B***A. cantonensis* (SYD.1) and *A. mackerrasae* (ANWC:N5721, P43/19-E) ITS2 rDNA. The ITS2 rDNA residue 56 that differentiates the two species is highlighted. The residue numbers correspond to the amplified region. Amplification primers mapped onto *A. cantonensis* (SYD.1) are highlighted with an arrow

Assay 2, targeting *cox*1 mtDNA, was used to detect *Angiostrongylus* spp. DNA in CSF samples of 61 dogs presenting with eosinophilic meningitis (DOG 1-61, of which 50 PCR-positive, 11 PCR-negative but antibody-positive, using PCR (Sears et al., 2021) in Lee et al. (2021)). Assay 2 in the present study returned 12 samples (12 of 50 known PCR-positive) with C_t_ values < 40 (C_t_ = 28.11–36.86) and melt curves corresponding to the *Angiostrongylus* spp. positive controls (Table 2). Unambiguous DNA sequences with > 98% percent identity to the reference *cox*1 DNA sequences for *A. cantonensis* (MK570631) and < 95% percent identity to *A. mackerrasae* (MN793157) were obtained for 10 (10/12) samples. For one sample (DOG55; C_t_ = 34.45) an incomplete DNA sequence was obtained, while the sequencing failed for another (DOG2; 36.86), likely due to low template DNA available for amplification (Table 2). In comparison with the reference *cox*1 haplotypes, the CSF sample from DOG53 had 100% sequence identity to *cox*1 of the SYD.1 reference sequence obtained from a wild rat (*Rattus norvegicus*) caught around the Taronga Park Zoological Gardens in Sydney 30 years ago (Červená et al., 2019). The remaining nine samples were 100% identical to each other (h2) and haplotype AC13 (KU532146) from Thailand. Haplotypes SYD.1 and h2 (AC13) had three distinct nucleotide differences in the 206-bp region amplified by Assay 2 (Fig. 2A). The *A. cantonensis cox*1 positive samples screened in the present study were collected between 2010 and 2019. The lone SYD.1 haplotype was collected from a dog from Sydney in 2012, while the AC13 haplotypes detected in the remaining dogs were found in dogs from both Sydney (*n* = 9) and Brisbane (*n* = 1).

**Table 2:** Summary of *Angiostrongylus* spp. identification in CSF samples from dogs with canine neuroangiostrongylosis

Sample ID	Real-time PCR Assay 2	Species (*cox*1)	Haplotype	ITS2	Species (ITS2)	Ultrasensitive real-time PCR^a^
DOG37	28.11	*A. cantonensis*	AC13	31.36	*A. cantonensis*	27.73
DOG57	31.33	*A. cantonensis*	AC13	33.01	*A. cantonensis*	33.76
DOG58	32.57	*A. cantonensis*	AC13	36.92	*A. cantonensis*	38.11
DOG23	33.16	*A. cantonensis*	AC13	38.21	*A. cantonensis*	31.07
DOG8	33.88	*A. cantonensis*	AC13	34.57	*A. cantonensis*	26.34
DOG49	34.10	*A. cantonensis*	AC13	38.71	fail	27.25
DOG53	34.20	*A. cantonensis*	SYD.1	35.55	*A. cantonensis*	25.84
DOG36	34.29	*A. cantonensis*	AC13	35.70	*A. cantonensis*	31.08
DOG55	34.45	*A. cantonensis*	fail	37.20	fail	33.08
DOG9	35.63	*A. cantonensis*	AC13	34.68	*A. cantonensis*	28.29
DOG52	35.87	*A. cantonensis*	AC13	37.12	fail	29.42
DOG2	36.86	fail	fail	neg		32.12
DOG11	neg			33.94	*A. cantonensis*	23.99
DOG34	neg			34.04	*A. cantonensis*	25.68
DOG50	neg			35.56	*A. cantonensis*	26.00
DOG16	neg			36.09	*A. cantonensis*	27.29
DOG12	neg			37.59	*A. cantonensis*	27.70
DOG25	neg			38.54	*A. cantonensis*	29.80
DOG43	neg			37.57	fail	26.71
DOG42	neg			38.15	fail	27.47
DOG61	neg			38.39	fail	33.94

a From Lee et al. (2021) using ultrasensitive real-time PCR (Sears et al., 2021).

### 3.4 Verification of *A. cantonensis* identity using a nuclear ITS2 marker in canine CSF samples from eastern Australia

To verify the species identity, we included a nuclear gene, ITS2, amplified using a previously developed assay (Fang et al., 2012). The ITS2 qPCR assay amplified DNA from both *A. cantonensis* and *A. mackerrasae*. Using this assay, *Angiostrongylus* DNA was detected in 20/61 of the CSF samples from dogs (32.8%) presenting with eosinophilic meningitis (50 PCR-positive, 11 PCR-negative but antibody-positive using the assay by Sears et al. (2021) in Lee et al. (2021)) *via* ITS2 qPCR with C_t_ values < 40 (C_t_ = 31.36–38.71; Table 2). Unambiguous *A. cantonensis* single nucleotide polymorphisms (SNP) in the DNA sequences were obtained for 14/20 ITS2 qPCR amplicons. The key ITS2 residue between reference *A. cantonensis* and *A. mackerrasae* is at position 56 of the ITS2 amplicon, where *A. cantonensis* has guanidine “G” while *A. mackerrasae* has adenosine “A” (Fig. 2B). All 14 ITS2 sequences obtained from dog CSF samples had “G” at the 56th residue, consistent with *A. cantonensis*.

## 4 Discussion

To the best of our knowledge, this is the first study that unequivocally demonstrates the presence of *A. cantonensis* partial *cox*1 mtDNA and ITS2 rDNA in CSF collected antemortem from dogs with NA confirming the long-held assumption that *A. cantonensis* is the causal agent of canine NA. Previously, a definitive diagnosis of *A. cantonensis* was only possible if larvae were obtained from CSF or at necropsy and thus available for morphological analysis. While we were unable to demonstrate the presence of *A. mackerrasae* in canine CSF in the present study, our results have insufficient numbers to rule out the possibility that dogs may also act as accidental hosts for this species, which is important in the context of Australia where both *A. cantonensis* and *A. mackerrasae* co-exist geographically (Lee et al., 2021).

Critically, the selected mtDNA marker gene (*cox*1) can discriminate both *A. cantonensis* (MK570631) and *A. mackerrasae* (MN793157) species with 9% pairwise difference, making it suitable for DNA amplification and subsequent sequence comparison. Mitochondrial DNA is ideal for species differentiation in samples with low amounts of template DNA because it is present in multiple copies per individual cell (Castellani et al., 2020). For these reasons, *cox*1 is often used for general ‘barcoding’ of living organisms (Hebert et al., 2003). The *cox*1 region has the additional advantage of being one of the most commonly used markers for the identification of *Angiostrongylus* spp. worldwide and thus a large number of sequences are already available for comparative studies and subsequent identification of *cox*1 haplotypes (Monte et al., 2012; Tokiwa et al., 2012; Moreira et al., 2013; Nakaya et al., 2013; Apichat et al., 2016; Rodpai et al., 2016; Eamsobhana et al., 2017; Dusitsittipon et al., 2018; Červená et al., 2019). Besides mtDNA, alternative markers include rDNA, i.e. ITS1 rDNA and ITS2 rDNA (Qvarnstrom et al., 2016; Lv et al., 2017). The use of a single marker that is maternally inherited, such as those on mtDNA, may be potentially deceiving when two closely related species coexist and present the opportunity for hybridisation and introgression (Ballard & Whitlock, 2004; Harrison & Larson, 2014; Chaudhry et al., 2015). Hybridisation has been hypothesised and suggested in an experimental study between *A. cantonensis* and *A. mackerrasae* (Bhaibulaya, 1974). Currently, however, no evidence suggests that hybridisation and introgression exist under field conditions in Australia, which is further supported by the results obtained in the present study where all *A. cantonensis cox*1 DNA-positive CSF samples had only ITS2 rDNA sequences matching *A. cantonensis*. Conversely, in Thailand, where *A. cantonensis* coexists with *Angiostrongylus malaysiensis*, a putative F1 hybrid has been identified using microsatellites analysis (Dusitsittipon et al., 2017, 2018).

Previous studies employing PCR primers targeting *cox*1 often used DNA isolated from *Angiostrongylus* nematode (adult or larvae) material and hence non-specific amplification of other parasite species or host DNA was not considered problematic (Monte et al., 2012; Tokiwa et al., 2012; Moreira et al., 2013; Okano et al., 2014; Apichat et al., 2016; Rodpai et al., 2016; Eamsobhana et al., 2017; Červená et al., 2019; Valentyne et al., 2020). In the present study, we used DNA isolated from canine CSF that lacked morphological evidence of the parasite, so needed to develop or adapt an assay able to amplify minimal quantities of *Angiostrongylus* spp. DNA (i.e. from fragments of the nematode exfoliated into CSF), while avoiding amplification of the abundant canine genomic and mitochondrial DNA (predominantly from nucleated eosinophils, the dominant cell fraction in CSF) (Lunn et al., 2012; Lee et al., 2021). This was achieved by *in silico* comparison of the selected primer sequences against the reference *cox*1 sequences of both *A. mackerrasae* and *A. cantonensis* and the adjustment of annealing temperatures to minimise amplification of host DNA. In order to facilitate this goal, we set three *in silico* criteria. First, primer sets needed to amplify a relatively short sequence (< 300 bp), in order to enable the amplification of potentially fragmented DNA, increase the limit of detection, and to allow adaptation of the assay for qPCR amplification (Dieffenbach et al., 1993). Secondly, the PCR amplicon needed to include sufficient variation to allow discrimination between *A. cantonensis* and *A. mackerrasae*. Thirdly, a maximum of three mismatches were permitted between the primer and *A. mackerrasae*/*A. cantonensis cox*1 sequences in order to ensure stringent annealing to the target DNA. The above *in silico* workflow streamlined the laboratory process into empirical steps, enabling us to optimise and select existing PCR assays that met the required criteria. The final adopted assay targets a region short enough to be amplified *via* qPCR, enabling streamlined sample throughput, but long enough to unambiguously discriminate between *A. cantonensis* and *A. mackerrasae* mtDNA sequences.

We confirmed the presence of *A. cantonensis* DNA in CSF samples from 11 dogs with canine NA based on the detection of *cox*1 mtDNA sequences and from 14 dogs based on ITS2 rDNA sequence (Lee et al., 2021). In total, there were 16 unique dogs confirmed to possess *A. cantonensis* DNA in their CSF (*cox*1 only, ITS2 rDNA only, or both), from an original cohort of 61 dogs with NA presenting with eosinophilic meningitis, of which 50 were originally considered qPCR-positive positive for *Angiostrongylus* DNA using an ultrasensitive qPCR assay targeting a repetitive element conserved within both *A. cantonensis* and *A. mackerrasae* DNA (Lee et al., 2021). Although unable to differentiate *Angiostrongylus* spp., the ultrasensitive qPCR is considered to be 100–1,000 times more sensitive than an existing diagnostic qPCR assay targeting ITS1 rDNA (Sears et al., 2021). Our ability to amplify DNA and discriminate partial *cox*1 sequences in 11/50 (22%) and 14/50 (28%) ITS2 sequences from available canine CSF samples exceeded our expectations, given the limited parasite DNA on offer for detection and the known superiority of the ultrasensitive qPCR assay. There was, however, no apparent relationship between the success of our *cox*1 amplification and detection using the ultrasensitive qPCR for *Angiostrongylus* spp. DNA (see Table 2). This difference may be related to the type of DNA on offer, given that *cox*1 is mitochondrial DNA while the repetitive region targeted by the ultrasensitive qPCR is nuclear DNA and hence, they are potentially under different constraints (e.g. fragmentation and digestion) within CSF.

As a consequence of our successful amplification of *A. cantonensis cox*1 mtDNA in canine CSF, a new *A. cantonensis* haplotype (AC13) was discovered in Australia. Previously, it was assumed that a single parasite introduction had facilitated the establishment of the Sydney *A. cantonensis* haplotype (SYD.1) along the eastern coastline of Australia (Červená et al., 2019). This initial incursion was always thought to have occurred in south-east Queensland rather than in Sydney, as the disease was seen in Brisbane about 20 years before it was observed in Sydney (Mackerras & Sandars, 1955; Alicata, 1991; Spratt, 2015). The results of the present study suggest that at least two, and potentially more, *A. cantonensis* introduction events have occurred in Australia. The existence of more than one haplotype outside the presumed original distribution within South East Asia is plausible as it has been previously demonstrated for specimens originating in Japan and Brazil (Monte et al., 2012; Tokiwa et al., 2012). The spread of *A. cantonensis* is facilitated by the introduction of either infected gastropod molluscs and/or rats (*Rattus* spp.), most likely as a result of translocation *via* cargo ships. Therefore, the existence of multiple *cox*1 haplotypes in Australia and beyond provides evidence of spread across the Pacific Ocean and South China Sea (Pien & Pien, 1999; Monte et al., 2012; Tokiwa et al., 2012, 2013; Červená et al., 2019).

Unlike the invasive *A. cantonensis*, *A. mackerrasae* is considered a local endemic species that evolved with the Australian rat species *Rattus fuscipes* and other rodents including *Melomys cervinipes* (Bhaibulaya, 1968). The absence of *A. mackerrasae* from the sequenced results demonstrates either a sampling or detection limitation of this study, or alternatively, supports the theory that *A. mackerrasae* does not normally infect dogs. Limited information is available concerning the genetic diversity of *Angiostrongylus* spp. in Australia. Further studies on *A. cantonensis* and *A. mackerrasae* in dogs, humans and wildlife using the ultrasensitive qPCR, alongside our *cox*1 assay to determine the species and haplotype from clinical samples (as in this study), are now feasible (Lee et al., 2021). Whether *A. mackerrasae* is capable of infecting other non-rodent hosts remains to be confirmed, either by direct experimental challenge, or *via* increased molecular surveillance. Australian wildlife species including possums, various parrot species and tawny frogmouths are considered sentinel animals for NA, with a morphologically identified specimen of *A. mackerrasae* found in a flying fox that contained first stage (L_1_) larvae in its faeces (Ma et al., 2013; Mackie et al., 2013). Despite these findings, the species of *Angiostrongylus* responsible for the bulk of disease in Australian wildlife has not yet been determined.

## 5 Conclusion

The confirmatory *cox*1 PCR assay adapted in our study enabled the unambiguous genetic identification of NA to the species level, thereby allowing differentiation between multiple *Angiostrongylus* spp. from antemortem canine CSF samples. While the assays were tested on canine CSF samples, they are readily applicable for use with CSF samples of non-canine origin (e.g. from human patients) or other material, including slugs and snails, as an auxiliary assay to the hyper-sensitive qPCR developed by Sears et al. (2021) or the ITS2 rDNA assay by Fang et al. (2012). Similarly, this approach can be considered for use in areas where multiple species of neurotropic *Angiostrongylus* spp. co-exist, including *A. cantonensis*, *A. malaysiensis* and *A. mackerrasae*.

## CRediT author statement

Jeevitheswara Mallaiyaraj: Conceptualization; Data curation; Formal analysis; Investigation; Methodology; Validation; Roles/Writing - original draft; Writing - review & editing. Nichola Calvani: Formal analysis; Methodology; Validation; Writing - review & editing. Rogan Lee: Investigation; Data curation; Methodology; Resources; Writing - review & editing. Richard Malik: Investigation; Data curation; Methodology; Resources; Writing - review & editing. Jan Šlapeta: Conceptualization; Data curation; Formal analysis; Funding acquisition; Investigation; Methodology; Project administration; Resources; Supervision; Validation; Writing - review & editing.

## Data availability

The nucleotide sequence data generated in this study were deposited in GenBank (NCBI) under the accession numbers MW898227-MW898236. Associated supplementary material is available at LabArchives (https://doi.org/10.25833/k7p1-m550).

## Declaration of competing interests

The authors declare that they have no known competing financial interests or personal relationships that could have appeared to influence the work reported in this paper.
